# Harnessing Genetics to Extend Lifespan and Healthspan: Current Progress and Future Directions

**DOI:** 10.7759/cureus.55495

**Published:** 2024-03-04

**Authors:** Paa Kwesi Ankrah, Enock D Mensah, Kwabena Dabie, Caleb Mensah, Benjamin Akangbe, Jonathan Essuman

**Affiliations:** 1 Division of Infectious Diseases, Duke University, Durham, USA; 2 Chemistry, Virginia Polytechnic Institute and State University, Blacksburg, USA; 3 Chemistry and Chemical Biology, University of New Mexico, Albuquerque, USA; 4 Translational Biology, Medicine and Health, Virginia Polytechnic Institute and State University, Blacksburg, USA; 5 Public Health, Georgia State University, Atlanta, USA; 6 School of Molecular Sciences, Arizona State University, Tempe, USA

**Keywords:** gerontology, gerontogenes, longevity, healthspan, lifespan

## Abstract

Aging is inevitable, but the lifespan (duration of life) and healthspan (healthy aging) vary greatly among individuals and across species. Unlocking the secrets behind these differences has captivated scientific curiosity for ages. This review presents relevant recent advances in genetics and cell biology that are shedding new light by untangling how subtle changes in conserved genes, pathways, and epigenetic factors influence organismal senescence and associated declines.

Biogerontology is a complex and rapidly growing field aimed at elucidating genetic modifications that extend lifespan and healthspan. This review explores gerontogenes, genes influencing lifespan and healthspan across species. Though subtle differences exist, long-lived individuals such as centenarians demonstrate extended healthspans, and numerous studies confirm the heritability of longevity/healthspan genes. Importantly, genes and gerontogenes are directly and indirectly involved in DNA repair, insulin/IGF-1 and mTOR signaling pathways, long non-coding RNAs, sirtuins, and heat shock proteins. The complex interactions between genetics and epigenetics are teased apart. While more research into optimizing healthspan is needed, conserved gerontogenes offer synergistic potential to forestall aging and age-related diseases. Understanding complex longevity genetics brings closer the goal of extending not only lifespan but quality years of life.

The primary aim of human Biogerontology is to enhance lifespan and healthspan, but the question remains: are current genetic modifications effectively promoting healthy aging? This article collates the advancements in gerontogenes that enhance lifespan and improve healthspan alongside their potential challenges.

## Introduction and background

The field of Biogerontology (biology of aging) is a complex and rapidly growing discipline. Genes that influence lifespan are broadly referred to as longevity genes, while all genes studied in Biogerontology are collectively known as gerontogenes. In ancient Greek and Roman times, demographic studies generally indicate a life expectancy between 20 and 35 years [1,2]. Despite the limitations of these earlier sources, it can be inferred that very few individuals during this time lived beyond 90 years [3]. While this outcome is considered low by 21st-century standards, it is noteworthy that individuals of the Greco-Roman era were less afflicted by aging-related diseases such as cancer, cardiovascular disorders, and multiorgan failures. This fact contradicts the idealized portrayal of lifespan extension, challenging the assumption that a longer lifespan naturally equates to a healthier life. The 21st century is ideally the era of aging. The world is getting older, and the numbers are consistently rising, with nearly 22% of the world population being ≥60 years old by the year 2050 [4]. Predictably, the cost of healthcare will rise due to numerous aging-associated diseases with immeasurable effects on quality of life. With most illnesses indicating underlying physiologic changes, delaying aging may help curb multiple health complications concurrently via modulating genetic pathways. As the mystery of aging becomes increasingly deciphered, the study of Biogerontology is founded on three key principles. First, lifespan is defined as the age at which death occurs, and the essential lifespan of a species marks the point beyond which there is a consistent decline in functional and physiological capabilities due to cellular aging, culminating in death [5,6]. The onset of aging is considered to begin after the essential lifespan is surpassed. Second, there is no single biological pathway or set of aging-associated genes (gerontogenes) identified as the sole cause of aging and death [7-9]. Lastly, the rate of aging varies among different species, individuals, and even within different organs and tissues of a single organism. This suggests that co-dependence and interconnected biological pathways across all levels play a crucial role in determining the overall lifespan of an individual [8,10,11].

Longevity and healthspan are often used interchangeably, yet they encompass distinct aspects. Longevity primarily centers around lifespan, the observed duration of an organism’s life [12], whereas healthspan focuses on healthy aging. Healthspan extends beyond the presence or absence of diseases or disease-susceptible alleles. It includes the development and maintenance of physical, physiological, and psychological capabilities [13] that contribute to overall well-being and delayed onset of age-associated diseases. Research indicates that individuals who live exceptionally long lives, such as centenarians, often experience a prolonged healthspan with minimal incidences of aging-related diseases such as cancer, cardiovascular disease, dementia, hypertension, and Alzheimer’s disease [14]. Healthy aging encompasses old age and good health with commendable body performance levels such as mobility and unharmed cognition [15]. Highlighting the significance of longevity and healthy aging genes is crucial, as numerous studies have demonstrated their heritability [15]. Notably, the likelihood of inheriting these genes increases with age, showing a probability of 0.48 in men and 0.33 in women, particularly in centenarians aged between 100 and 109 years [16]. Further evidence of the association between inherited longevity genes and increased age has been observed in extensive studies involving over 20,000 Scandinavian twins [17], Icelanders [18], and other centenarian populations [19,20]. Additionally, comprehensive studies like the New England Centenarian Study [21], the Leiden Longevity Study [22], and the Long Life Family Study [23] have compared the offspring of long-lived individuals to contemporaneous controls. These offspring not only exhibited longer average lifespans but also displayed various healthy aging characteristics, including beneficial lipid profiles [24], a low rate of cardiovascular and metabolic diseases [25], and a lower prevalence of hypertension compared to their age-matched controls [24,26]. In recent decades, Biogerontology has undergone a significant transformation, shifting from limited access to aging pathways to advanced genetic modifications that can extend lifespan. Contemporary Biogerontology focuses on how these longevity genes impact healthspan. It is noteworthy that healthspan can be significantly altered, either enhanced or deteriorated, due to interventions targeting lifespan, often leading to increased morbidity [26].

**Figure 1:**
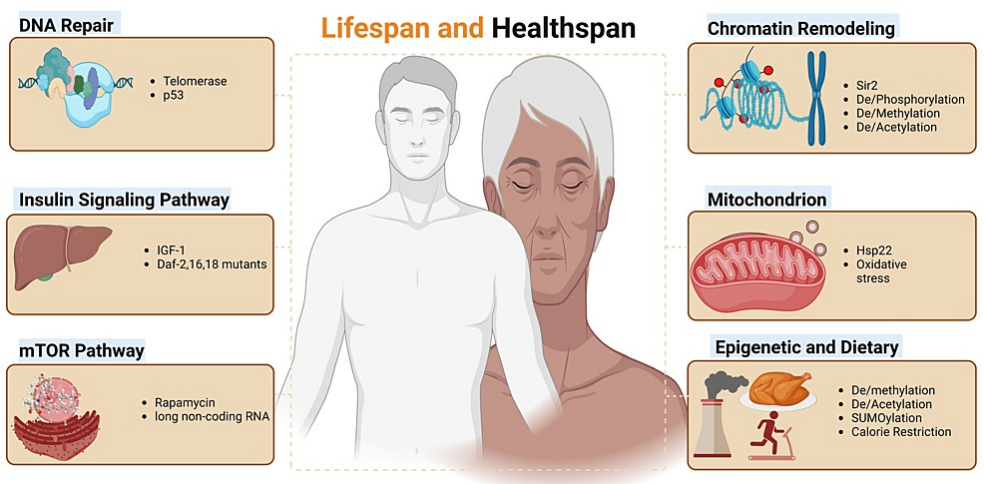
Genetic, epigenetic, and environmental factors influencing lifespan and healthspan. Image generated using BioRender.com.

The relationship between an organism’s lifespan and the maintenance of cellular youthfulness reveals a complex interconnection of pathways. While there is no single, definite hypothesis to explain the rate of aging, identified contributing factors include genetics, a combination of epigenetics, physiological factors, and lifestyle choices. Four broad groups of genes are conserved across various kingdoms and have been identified to influence aging. These include genes responsible for (i) DNA repair enzymes, (ii) proteins in the insulin signaling pathway, (iii) mechanistic target of rapamycin (mTOR) signaling pathway proteins (translation regulators), and (iv) chromatin remodeling enzymes. Further studies extensively explore the role of mitochondrial heat shock protein genes, such as Hsp22, and various epigenetic and environmental factors that contribute to aging (Figure 1).

## Review

DNA repair enzymes

Possessing efficient DNA repair enzymes resistant to mutations enhances longevity by mitigating the effects of aging-related “wear and tear.” The wear-and-tear hypothesis proposes that the efficiency of cell division and enzymatic functions decline with age [27,28]. Mutations in DNA repair enzymes, even in younger individuals, can lead to premature aging conditions, such as progerias [29,30]. The transcription factor *p53* plays a critical role in regulating cell division. It arrests the cell cycle and activates DNA repair enzymes [31,32]. The pleiotropic effect of *p53 *allows multi-level regulation from diverse factors, demonstrated with mouse double minute 2 (*Mdm2*) and *Mdm4* [33]. In the absence or damage to telomeres, *p53 *becomes active, halting DNA replication and facilitating DNA repair. The role of telomeres and telomerase activity in cellular immortality has been a long-standing proposition [34]. The telomerase enzyme replenishes telomere length after each cell cycle. Studies have shown that telomere lengths are longer in children than adults, supporting telomeres’ role in aging [34]. Again, a study using transgenic mice, replacing mouse telomerase RNA (*mTR*) with neomycin-resistant genes decreased telomere length and telomerase activity with age [35,36]. Similarly, older zebrafish exhibited shorter telomeric repeats (TTAGGG)n in the terminal restriction fragment (TRF) compared to their younger counterparts [37]. Reduction in the telomeric repeats (TTAGGG)n sequence has been associated with chromosomal instability, increased aneuploidy, and a higher incidence of cell-to-cell fusions [38]. Similarly, cells harboring a mouse telomerase null mutation exhibited no telomerase activity and, consequently, the least lifespan [39]. The length of telomeric repeats which cap chromosomal ends is inversely proportional to aging. Premature aging disorders, such as progeria, spleen atrophy, disrupted germinal centers, and reduced proliferation in bone marrow and neural stem cells, have been associated with a lack of telomerase and a deleted or nonfunctional telomerase gene [40,41]. This association led to the hypothesis that extending telomeric repeats might enhance healthspan. However, attempts to maintain longer telomeric stability present challenges, as they often seed tumorigenesis due to uncontrolled cellular division [42]. Present methods for measuring telomere length, including TRF analysis and quantitative polymerase chain reaction, are subject to minor margins of error that affect the accuracy of telomere length results. Despite the experimental and logistical reliability of these established techniques, their inherent drawbacks can be addressed by more advanced methodologies such as single telomere length analysis and telomere shortest length assay (TESLA), albeit at increased financial and labor costs [43]. Telomere length exhibits variations of approximately ±2-4% per month, challenging the prevailing assumption of consistent telomeric attrition with age [44].

Mechanistic target of rapamycin pathway

**Figure 2:**
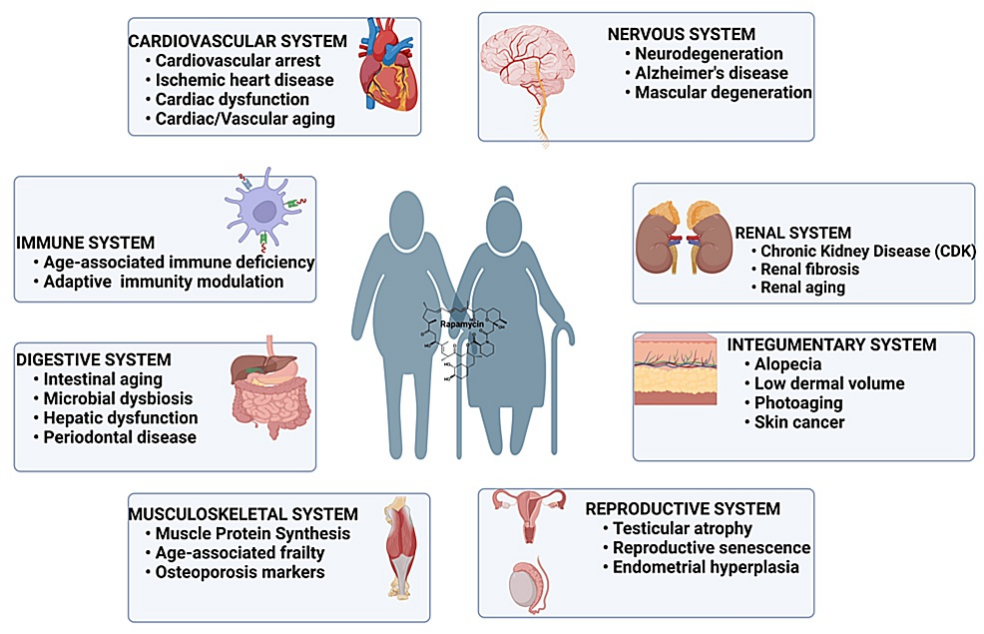
General role of rapamycin in sustaining organ health. Image generated using BioRender.com.

Altering genes in the mTORC1 pathway directly impacts cellular longevity [45]. When activated, the insulin signaling pathway upregulates the activity of the mTORC1 pathway and inhibits the *FoxO* transcription factor [45,46]. In the mTORC1 pathway, the protein kinase complex mTORC1 stimulates the conversion of mRNA into essential proteins and enzymes in response to nutrients and hormones [47]. Inhibiting mTORC1 interferes with insulin signal transmission, leading to reduced metabolism, slowed aging, and a decline in age-related conditions such as cognitive dysfunction [48,49]. Since the discovery of rapamycin as an antibiotic in 1975, it has been extensively studied for its potential to extend lifespan [50]. Studies show that rapamycin, in combination with FK506-binding proteins, downregulates genes in the mTOR pathway and is evident in yeast [51], nematodes [52], rotifers [53], *drosophila *[54], and mouse models [55,56]. Interestingly, rapamycin has a sexually dimorphic effect on mTOR signaling with varying mean healthspan extension in females than males in different research models [52,57,58]. Growing evidence indicates that rapamycin contributes to an improved healthspan, as seen through delayed aging of organs (Figure 2) and reduced incidence of disease conditions, including liver degeneration, ovarian aging, heart abnormalities, and benign tumors in the adrenal gland [59,60]. Mechanistically, rapamycin improves healthspan via inhibition of the mTOR pathway, which impacts multiple biological processes such as apoptosis, inflammation, cell growth, and metabolism [60,61]. Rapamycin specifically inhibits the mTORC1 signaling pathway [62] to enhance healthspan through delayed aging in kidneys, liver, ovaries, intestines, respiratory, circulatory, integumentary, and immune systems. A major setback in rapamycin clinical administration is the simultaneous inhibition of mTORC2 signaling while blocking the mTORC1 pathway [62]. This off-target effect induces adverse effects on metabolic and immunologic functions and can be prevented by selectively inhibiting mTORC1 with rapamycin derivatives and structural analogs. An extensive study introduced the importance of long noncoding RNAs as essential regulators of mTOR signaling, in general, to improve lifespan expectancy and decline onset of aging-related disorders and cancer in humans [63]. Currently, rapamycin is a great candidate for both lifespan and healthspan extension with extensive research and clinical studies.

Insulin and insulin-like growth factor 1 signaling pathways

Similar to DNA repair enzymes, genes within the insulin signaling pathway exhibit a functionally conserved role in aging across various species. Downregulation of these genes indicates limited food availability, leading to a decline in metabolism and an enhanced synthesis of free radical scavenging enzymes via the upregulation of the *FoxO/DAF-16* transcription factor [64-66]. *FoxO *is crucial to the immortality of hydra and a conserved longevity regulator operating downstream of insulin signaling pathways of several species in the animal kingdom. Several reports indicate that the IGF-1 receptor regulates lifespan and oxidative stress in mice [67,68]. Upon modification of different exons in the IGF-1 gene and feeding mice ad libitum, heterozygous IGF-1 mice lived longer than the wild type [69,70]. In animal models, such as *Caenorhabditis elegans* and *Drosophila*, reduced insulin signaling has been linked to an extended lifespan [71,72]. As aging broadly depicts a decline in physical activities, studies involving the maximum velocity of *C. elegans* revealed that the daf-2 mutant (E1370) exhibited a higher maximum velocity, approximately twice that of the wild type [73]. Notably, the daf-2 mutant/insulin/IGF-1 receptor exhibits pleiotropic effects by exponentially enhancing lifespan and immunity while diminishing reproduction and motility [74]. Loss-of-function mutation in daf-18(nr2037), encoding phosphatase and tensin homolog (*PTEN*), is essential to the extended lifespan of daf-2 mutants [75]. *PTEN *dephosphorylates phosphatidylinositol 3,4,5-trisphosphate to phosphatidylinositol 4,5-bisphosphate [76]. Despite disorders in insulin signaling causing conditions such as type 1 and 2 diabetes and neuronal dysfunction due to metabolic stress [77], they have been associated with extended lifespans in humans [78], *C. elegans *[64], and *Drosophila melanogaster* [79]. A shared trait among these organisms is the reduction in serum IGF-1, associated with the insulin/IGF signaling cascade. However, mutagens in ingested food and free radicals such as hydroxyl and superoxide radicals formed as metabolic by-products accumulate to induce age-related diseases. Reduced IGF-1 signaling by deleting the IGF-1 receptor decreases metabolism and eventually reduces the overall cell senescence caused by oxidative stress [77]. Furthermore, due to higher metabolic rates in males than females, the overall impact of lifespan extension varies between genders [80]. This conservation of insulin signaling pathways and their influence on aging highlights a fundamental aspect of biology that transcends species, offering potential insights for human aging and longevity research.

Chromatin remodeling

Genes encoding proteins to modify chromatin structure influence aging and aging-associated diseases by regulating the spatial expression of genes during development. Chromatin modification techniques include but are not limited to methylation/demethylation, phosphorylation, acetylation/deacetylation, and sumoylation. Geneticist Amar Klar first described sirtuins in the 1970s when he discovered the silent information regulators II (*Sir2*) gene in *Saccharomyces cerevisiae* cells. However, the only study demonstrating an effect on lifestyle was published 20 years later in 1991 by Leonard P. Guarante [81]. Sirtuins belong to the class of deacetylases that help coordinate DNA repair, cell survival, healthy aging, development, apoptosis, and metabolic control [82]. The sirtuin gene, known for its anti-aging properties and encoding proteins involved in chromatin silencing through histone deacetylation, is found across the eukaryotic kingdoms [83]. As growth occurs, sirtuin gene products, such as enzymes and proteins, are re-purposed to repair single- and double-stranded DNA breaks at the expense of chromatin silencing [82]. Consequently, genes that were initially silenced become active as aging progresses. Cognitive decline, associated with the mammalian aging syndrome, can be mitigated by upregulating DNA methylation and histone (H4K12) acetylation in the brain’s frontal lobes [83]. Sirtuins comprise seven regulatory proteins for metabolism, antioxidant protection, and cell cycle regulation. Deletion of the *Sir2 *gene, responsible for the expression of sirtuins, shortens the lifespan of *S. cerevisiae* and has, therefore, become an important focus of research on the aging process [84]. Contemporary methodologies associated with chromatin analysis are both high-throughput and labor-intensive, necessitating the use of chromatin immunoprecipitation, antibody staining, microscopy, and, at times, sequencing. The experimental workflows employed in chromatin studies can introduce background noise and variability and often permit only relative quantification [85].

In conjunction with sirtuins, polyphenols such as butein, fisetin, and resveratrol have been shown to extend yeast lifespan by 31%, 55%, and 70%, respectively, also contributing to an increase in the maximum lifespan for each of these activators [86]. Research has highlighted the potency of resveratrol to delay the onset of aging-associated diseases by mimicking calorie restriction [87]. Over time, the homologous recombination between the ribosomal DNA repeats, forming a circular, autonomously replicating extrachromosomal DNA, which is toxic in old cells. Resveratrol increases the activity of yeast *Sir2 *to prevent the formation of extrachromosomal structures [88]. The synergistic increment in lifespan in the presence of polyphenols is a culmination of antioxidant, metal ion chelating, and free radical scavenging roles of polyphenols and the chromatin silencing effect of sirtuin via histone deacetylation. Invariably, no observable increments in lifespan are noticed in *Sir2 *null mutants in *S. cerevisiae *[89] and humans [90]. Hence, *Sir2 *primarily regulates longevity, while polyphenols directly stimulate *Sir2 *activity in vivo.

Epigenetics and diet

**Figure 3:**
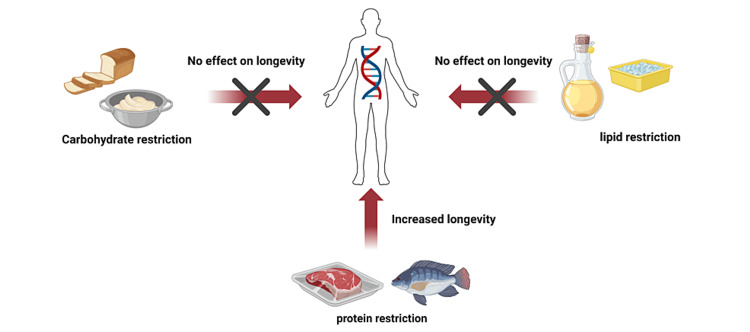
Effects of dietary restriction of major food nutrients on longevity. Image generated using BioRender.com.

In addition to genetic determinants, the effects of epigenetic factors influence the overall lifespan of an organism. Susceptibility to errors during cell division cycles and possible activation of oncogenes increase with prolonged cell divisions, making aging a protective mechanism against tumorigenesis [91]. The methylation of CpG islands occurs under several conditions such as genetic, dietary, and exposure to xenobiotics and mutagens. While Ames dwarf mice have thrice fewer CpG islands than wildtype, caloric restriction prevents demethylation at hypomethylated regions and enhances methylation of hypermethylated loci than rapamycin [92]. Thus, the epigenetic influence on the aging process is far more convoluted and is species-specific. Therefore, studies involving cell longevity and healthspan involve cautious consideration of the numerous ongoing, interrelated pathways, cell divisions, and reactions. Dietary intake has strong correlations with aging. A highly inflammatory diet dwindles the body’s repair potential and eventually speeds up aging [93]. This reduction in the repair potential arises from the continuous disruption of cell types responsible for the repair of inflammation resulting from tissue injury [94]. Dietary restriction (caloric reduction) is known to delay aging and the onset of diseases as well as enhance longevity in most organisms [95]. Dietary enhancement of longevity is best achieved by a combination of low protein/high carbohydrate food rations (Figure 3) [96] as low protein/high carbohydrates inhibit the mTORC1 pathway, enhance thrombospondin signaling pathway, and suppress the production of reactive oxygen species by the mitochondrion as opposed to the lower intake of carbohydrates and fats [97]. This reduction in reactive oxygen species coupled with a decrease in metabolic rate has long been regarded as the means through which dietary restriction may prolong lifespan. Several studies have cited caloric reduction as important in preventing the signs of aging, such as telomere deterioration, epigenetic changes, genome instability, mitochondrion inefficiency, and cellular senescence [42,98,99]. Moreover, caloric reduction effectively limits age-associated alterations in DNA methylations in vital organs [100] and delays age-related decline in mitochondrial biogenesis and function [101].

Mitochondrion and aging

Aging is generally associated with increased oxidative stress and declining mitochondrial efficiency. The mitochondrial gene encoding heat shock proteins, Hsp, has garnered significant interest in anti-aging studies. A study by Lagunas-Rangel highlighted the influence of G protein-coupled receptors on lifespan using animal and human models [102]. Given mitochondria’s sensitivity to reactive oxygen species, the presence of genes that produce antioxidants within mitochondria is essential. The overexpression of Hsp22 has been reported to protect mitochondrial proteins within motoneurons and extend the lifespan of *D.* *melanogaster *[103]. Hsp22 genes contribute to extending the lifespan in *D. melanogaster *by predominantly guarding against oxidative stress. Studies have highlighted the functional roles of mitochondrial Hsp22 in lifespan extension and oxidative stress resistance in *Drosophila *[104]. Conversely, overexpression of Hsp22 yielded a reduced lifespan in mouse models due to myocardial hypertrophy [105]. An optimal mitochondrial function stems from good protein quality. The Hsp22 proteins act as molecular chaperones against ruined proteins, as unchecked damaged proteins gradually amass into toxic aggregates with further deteriorating effects on mitochondrial integrity. Hsp22 concentration is an aging biomarker in *D. melanogaster* and predicts the remaining lifespan. Ultimately, Hsp22 increases longevity by chaperoning damaged proteins and improving stress resistance.

## Conclusions

To enhance the applicability of these models to human research, efforts should concentrate on identifying study metrics that accurately represent the unique physiological and pathological aspects of aging in different species. The gerontogenes encoding telomerase/telomeric repeats, insulin-like growth factor receptor 1, Sir2, and mitochondrial heat shock proteins (Hsp22) enhance longevity, while daf-2 gene mutants and downregulation of mTOR signaling genes by rapamycin enhance both longevity and healthspan. Healthspan is a complex, multi-metric analysis of functional and physiologic efficiencies of organs in different organisms. More studies are needed to streamline the healthspan conception and the best multi-metrics for a specific anti-aging model organism. Moreover, these conserved gerontogenes influencing longevity and healthspan are functionally nonexclusive; hence, understanding the synergistic efforts of two or more gerontogenes can introduce novel techniques to minimize geriatric syndrome while maintaining healthy cells.
